# Supplementary material for: Longitudinal liquid biopsy identifies an early predictive biomarker of immune checkpoint blockade response in head and neck squamous cell carcinoma
Source: Nat Commun. 2025 Sep 1;16:8161. doi: 10.1038/s41467-025-63538-4 (PMC12402333; doi:10.1038/s41467-025-63538-4)
Supplement: Supplementary file 1 — Supplementary Information [file 41467_2025_63538_MOESM1_ESM.pdf]

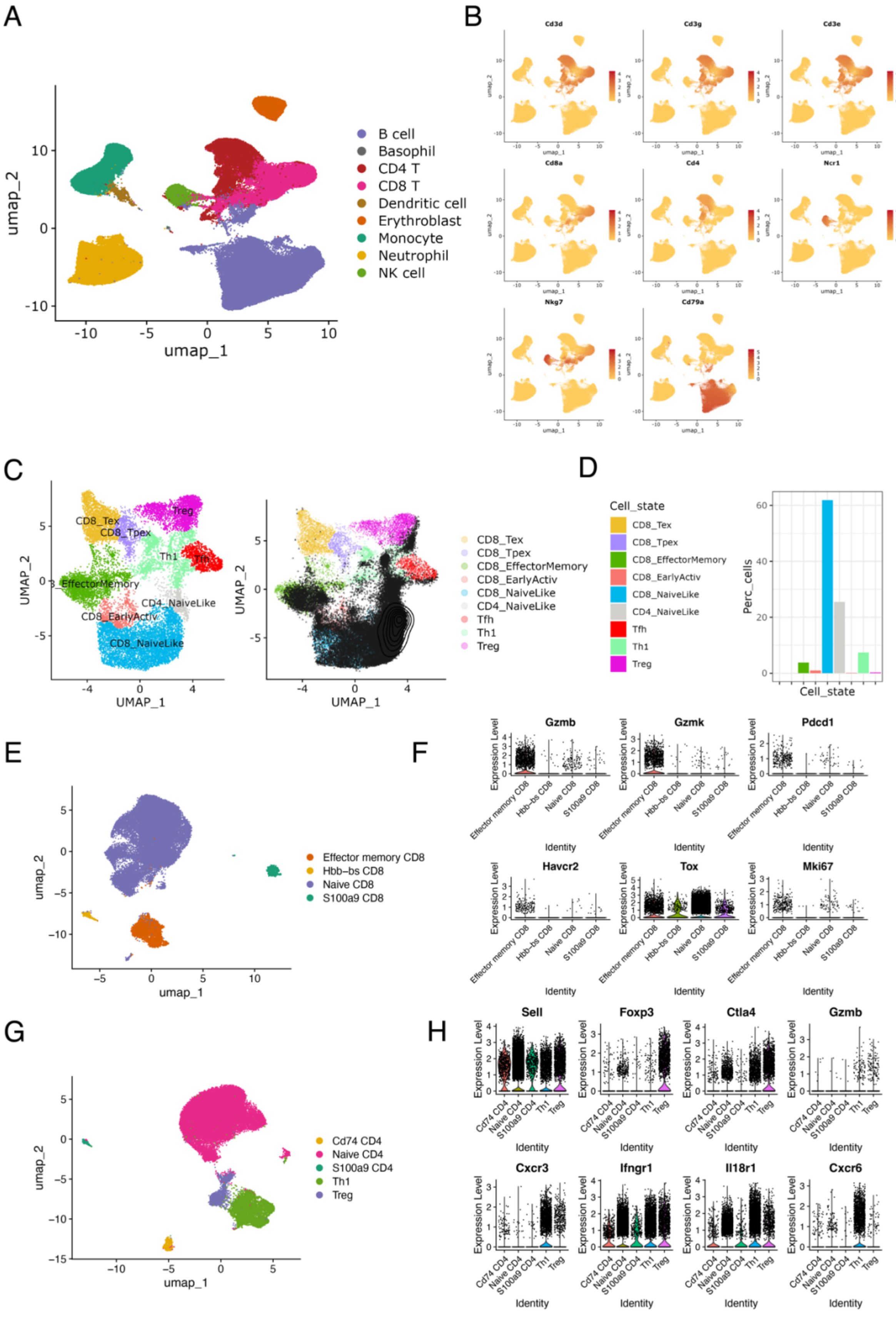
A
umap_2
umap_1
B cell
Basophil
CD4 T
CD8 T
Dendritic cell
Erythroblast
Monocyte
Neutrophil
NK cell
B
Cd3d
Cd3g
Cd3e
Cd8a
Cd4
Ncr1
Nkg7
Cd79a
C
CD8_Tex
CD8_Tpex
Treg
Th1
Tfh
CD4_NaiveLike
CD8_EarlyActiv
CD8_NaiveLike
UMAP_2
UMAP_1
CD8_EffectorMemory
D
Cell_state
Perc_cells
E
Effector memory CD8
Hbb−bs CD8
Naive CD8
S100a9 CD8
umap_2
umap_1
F
Gzmb
Gzmk
Pdcd1
Havcr2
Tox
Mki67
Expression Level
Identity
G
Cd74 CD4
Naive CD4
S100a9 CD4
Th1
Treg
H
Sell
Foxp3
Ctla4
Gzmb
Cxcr3
Ifngr1
Il18r1
Cxcr6

Supplementary Figure 1. Identification of cell types in mouse blood single-cell RNA-seq data.

A. UMAP embedding of cells in mouse blood, illustrating the overall distribution of cell types. B. Expression of marker genes corresponding to the major cell types identified in the single-cell data. C. UMAP embedding of reference T cells (left panel) and the in-house single-cell data (right panel), with in-house data represented by black dots. D. Distribution of T cell subpopulation abundance in the in-house single-cell data. E. UMAP embedding showing subclusters of total CD8+ T cells. F. Expression of marker genes specific to CD8+ T cell subpopulations. G. UMAP embedding showing subclusters of total CD4+ T cells. H. Expression of marker genes specific to CD4+ T cell subpopulations.

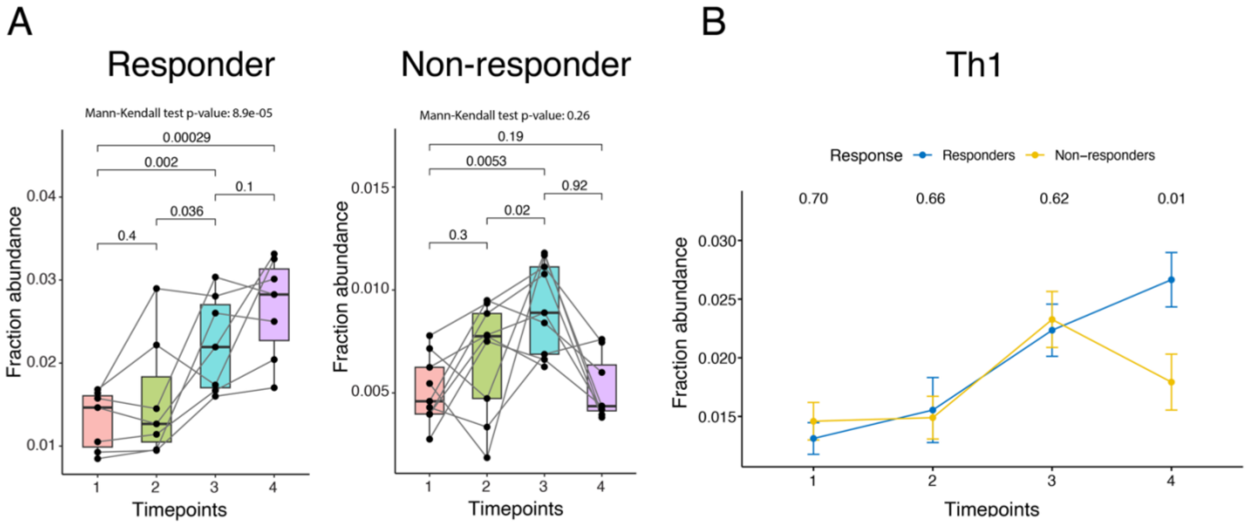

Supplementary Figure 2. Temporal changes in the abundance of CD4+ T cell subclusters following ICB treatment.

A. Dynamic changes in the abundance of Type 1 T helper (Th1) in responders (n = 7) and non-responders (n = 9) following ICB treatment. B. Abundance differences of in Th1 cell abundance changes between responders (n = 7) and non-responders (n = 9). Statistical significance was calculated using a one-tailed Wilcoxon test. In box plots (panel A), the center line indicates the median; the box spans the interquartile range (IQR, 25th to 75th percentile); whiskers extend to values within 1.5× IQR from the quartiles; and each dot represents one biological replicate (a single mouse). In panels B, dots represent the mean, and error bars indicate the standard error of the mean (±SEM). The unit of study is the individual mouse. Statistical significance was assessed using a one-tailed Wilcoxon rank-sum test unless otherwise noted. The Mann-Kendall test was used to evaluate monotonic changes across time points. For all panels, the X-axis represents time points, and the Y-axis represents the fraction of cells out of the total measured. Source data are provided as a Source Data file.

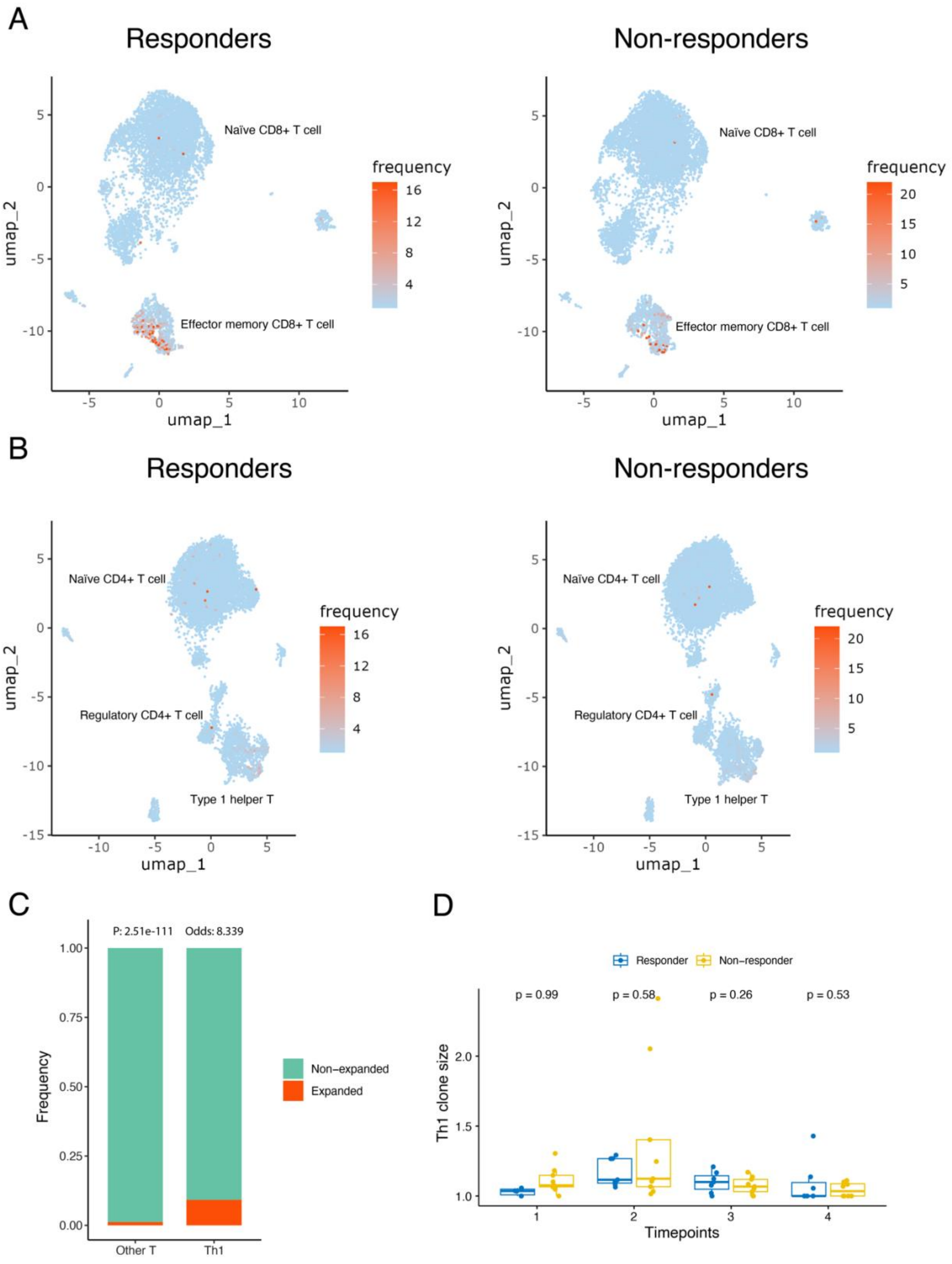
A
Responders
Non-responders
Naïve CD8+ T cell
Effector memory CD8+ T cell
frequency
umap_1
umap_2
B
Responders
Non-responders
Naïve CD4+ T cell
Regulatory CD4+ T cell
Type 1 helper T
frequency
umap_1
umap_2
C
P: 2.51e-111
Odds: 8.339
Frequency
Non−expanded
Expanded
Other T
Th1
D
Responder
Non−responder
p = 0.99
p = 0.58
p = 0.26
p = 0.53
Th1 clone size
Timepoints

Supplementary Figure 3. Clonal size distribution of T cells between responders and non-responders.

A. UMAP of CD8+ T cells in responders and non-responders, with each dot representing a single cell. Cells are colored according to their clone size, highlighting differences in clonal expansion. B. UMAP of CD4+ T cells in responders and non-responders, with each dot representing a single cell. Clone sizes are indicated by color, emphasizing clonal diversity within the CD4+ T cell population. C. Distribution of expanded CD4+ T cell clones (clone size ≥2, represented by red bars) and non-expanded clones (clone size =1, represented by green bars) between Th1 cells and other CD4+ T cells. P-value and odds ratio were calculated using Fisher's exact test. D. Distribution of CD4+ T cell clone sizes between responders and non-responders across four timepoints. Statistical significance was determined using a one-tailed Wilcoxon test. Each dot represents one sample. Source data are provided as a Source Data file.

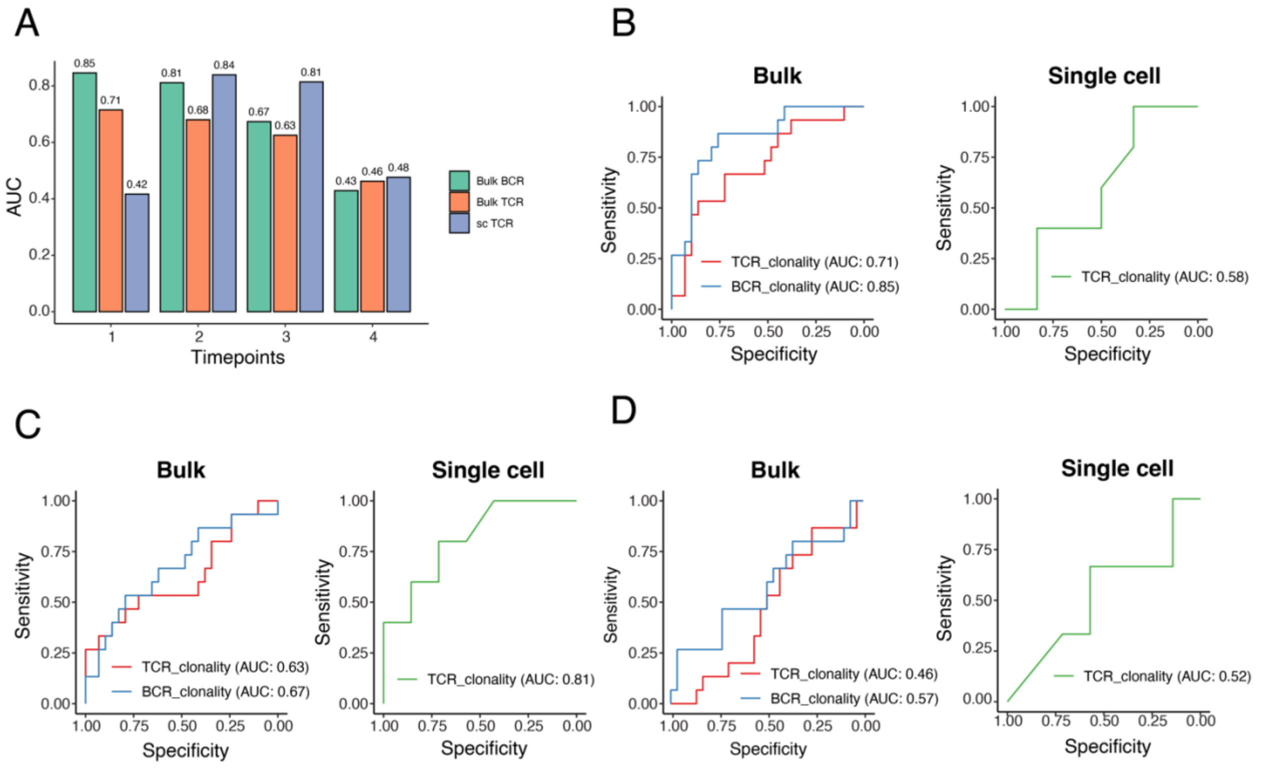

Supplementary Figure 4. Prediction of ICB response using T cell and B cell clonal expansion in a mouse model.

A. Area Under the Curve (AUC) values for predicting ICB response based on the bulk B cell clonal expansion index, bulk T cell clonal expansion index, and single-cell T cell clonal expansion index across four time points. B-D. Receiver Operating Characteristic (ROC) curves and corresponding AUC values for ICB response prediction using the clonal expansion index of T and B cells at time point 1 (B), time point 3 (C), and time point 4 (D). Source data are provided as a Source Data file.

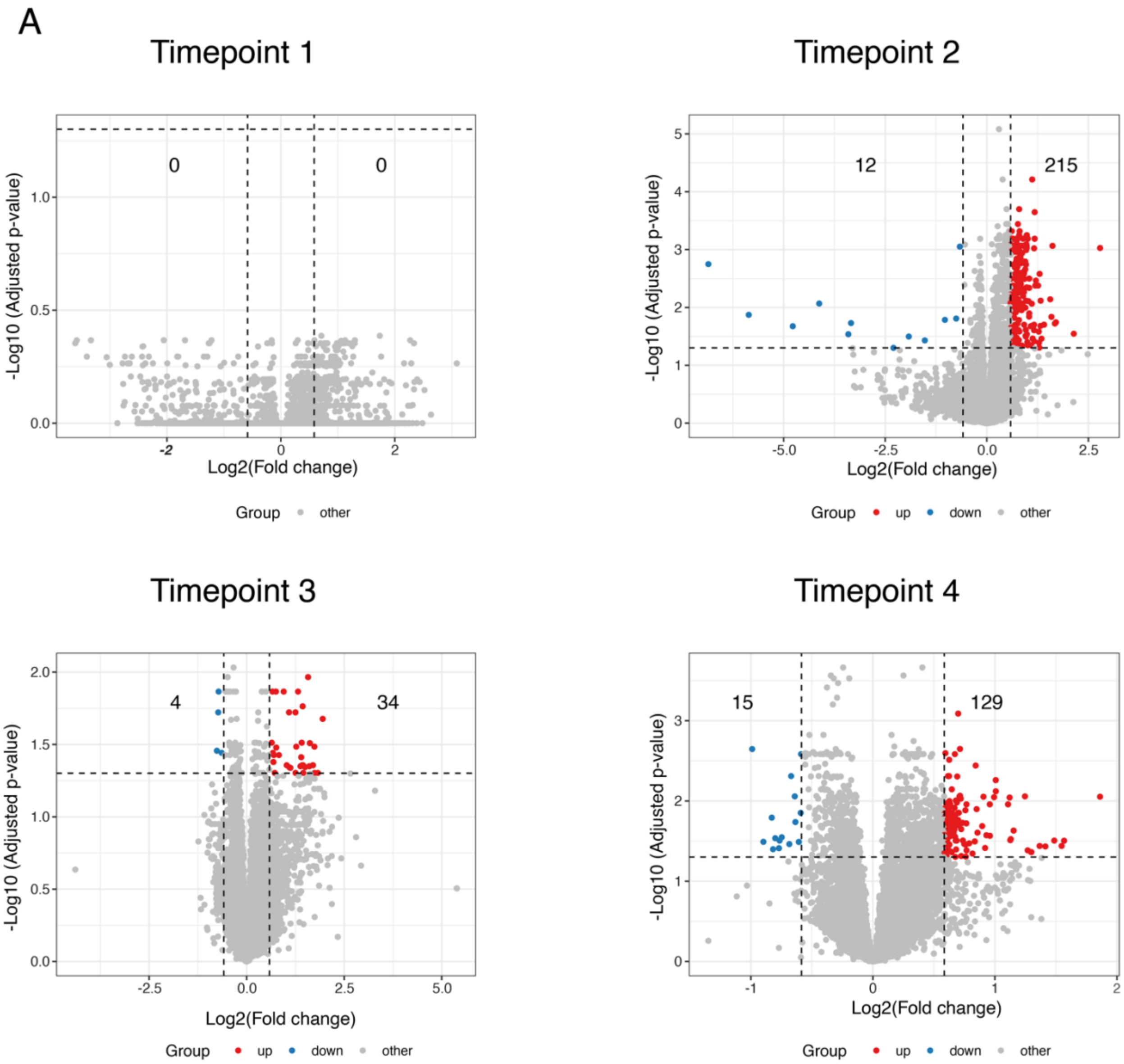

Supplementary Figure 5. Identification of differentially expressed genes between responders and non-responders at each time point.

A. The X-axis represents the $\log_2$-transformed fold change between responders and non-responders. The Y-axis represents the negative $\log_{10}$-transformed adjusted p-value. Upregulated genes (fold change ≥ 1.5, adjusted p-value ≤ 0.05) are colored red, while downregulated genes (fold change ≤ -1.5, adjusted p-value ≤ 0.05) are colored blue.

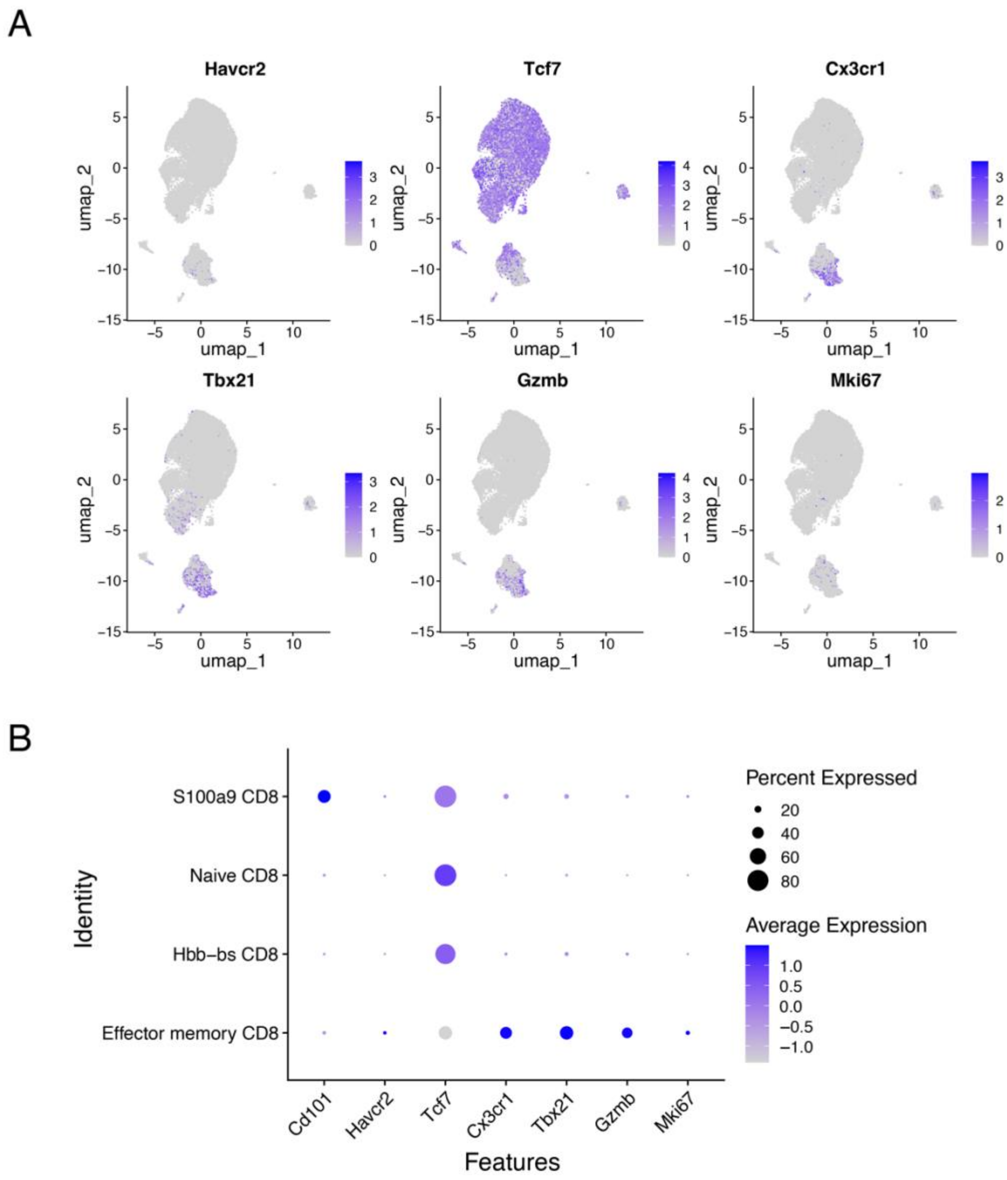

Supplementary Figure 6. Expression of transitional CD8⁺ T cell signatures in the effector memory T cell subpopulation

A. UMAP plot showing the expression of genes associated with transitional $CD8^+$ T cells within the $CD8^+$ T cell population. B. Heatmap illustrating the high expression of transitional $CD8^+$ T cell markers specifically within the effector memory $CD8^+$ T cell subpopulation.

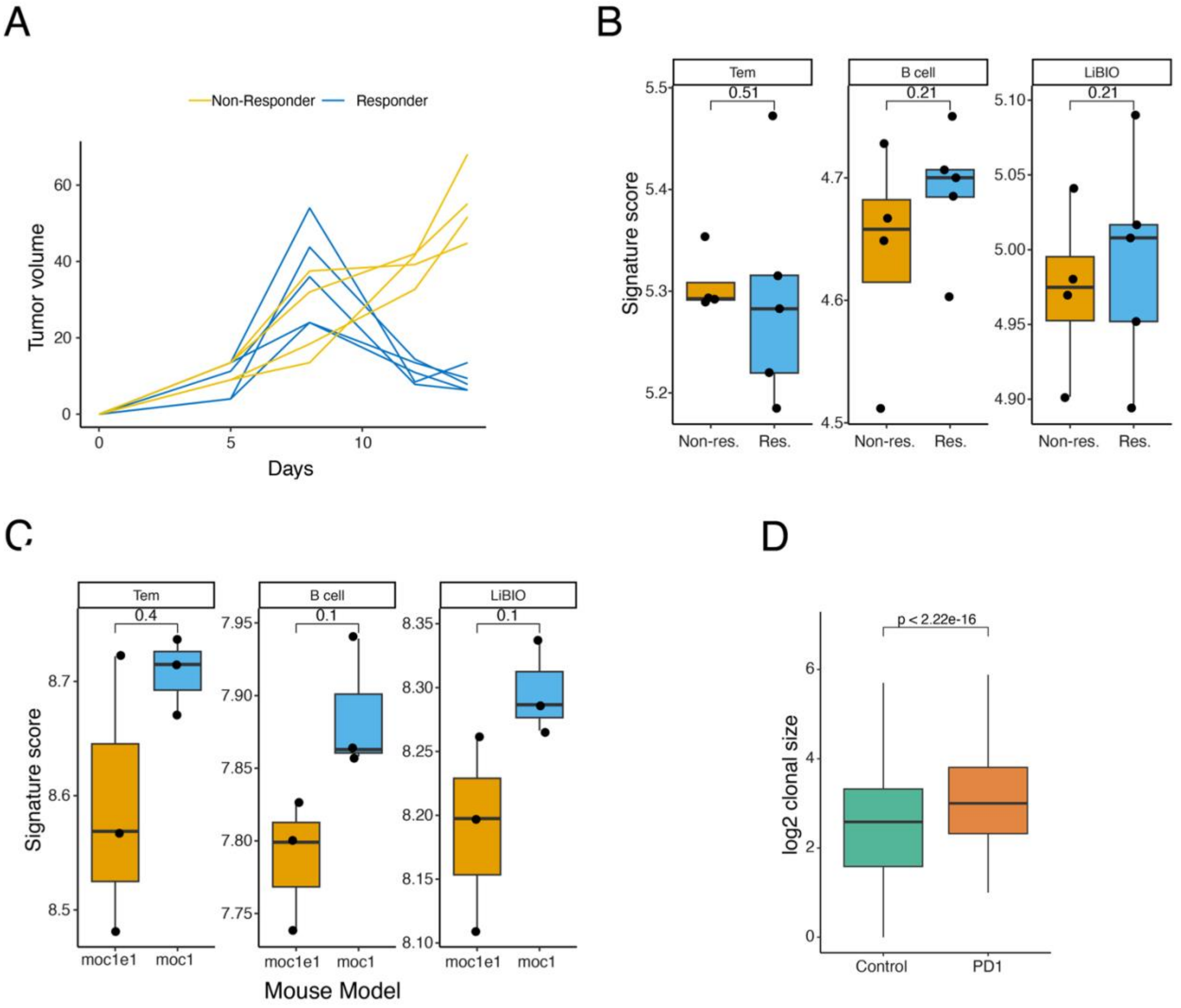

Supplementary Figure 7. Distribution of immune signature scores and T cell clonal expansion in ICB-sensitive and ICB-resistant mice.

A. Tumor volume trajectories in responder (blue lines) and non-responder (yellow lines) mice following anti-PD-1 treatment. B. Effector memory T cell (T_{em}), B cell, and LiBIO scores in responder versus non-responder mice based on in-house tumor RNA-seq data. C. T_{em}, B cell, and LiBIO scores in ICB-sensitive (MOC1) and ICB-resistant (MOCe1) mouse models from a publicly available dataset. D. T cell clonal expansion in ICB-sensitive mice (MOC1), represented as $\log_2$-transformed clonal sizes before (green boxes) and after (orange boxes) anti-PD-1 treatment. In box plots, the center line indicates the median; the box spans the interquartile range (IQR, 25th to 75th percentile); whiskers extend to values within 1.5× IQR from the quartiles; and each dot represents one biological replicate (a single mouse). Statistical significance was evaluated using the two-tailed Wilcoxon rank-sum test.

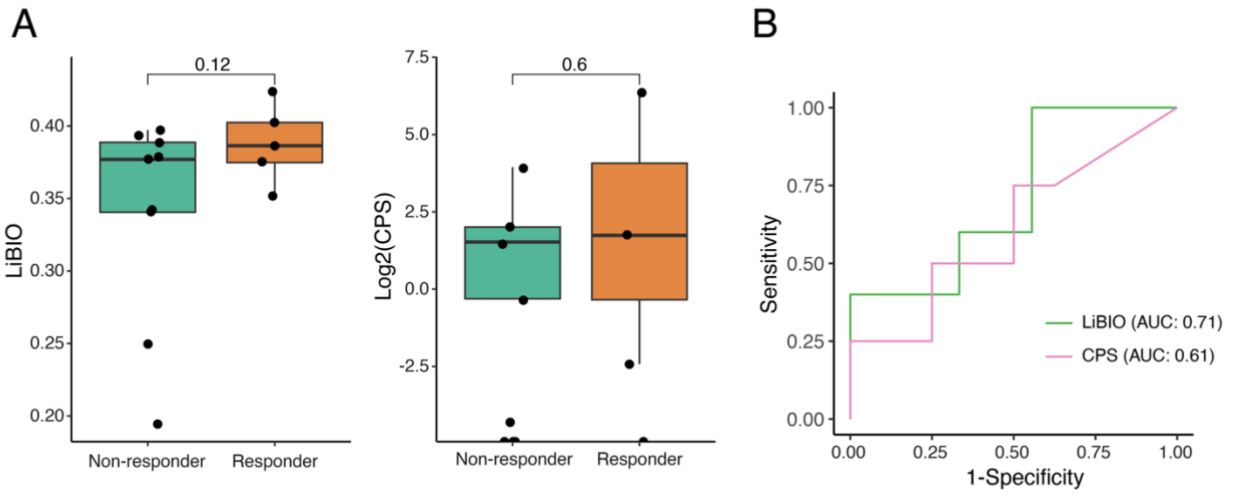

Supplementary Figure 8. Prediction of ICB response in HNSCC using the LiBIO and CPS scores.

A. Boxplots showing the distribution of LiBIO and estimated CPS scores (based on CD274 expression) between ICB responders and non-responders. In box plots, the center line indicates the median; the box spans the interquartile range (IQR, 25th to 75th percentile); whiskers extend to values within 1.5× IQR from the quartiles; and each dot represents one biological replicate (a single patient). Statistical comparisons were made using two-tailed Wilcoxon rank-sum tests. B. ROC curves comparing the predictive performance of the LiBIO score and transcriptomic based CPS score. The LiBIO score demonstrated superior accuracy (higher AUC), indicating its potential as a more robust predictor of ICB response. Source data are provided as a Source Data file.

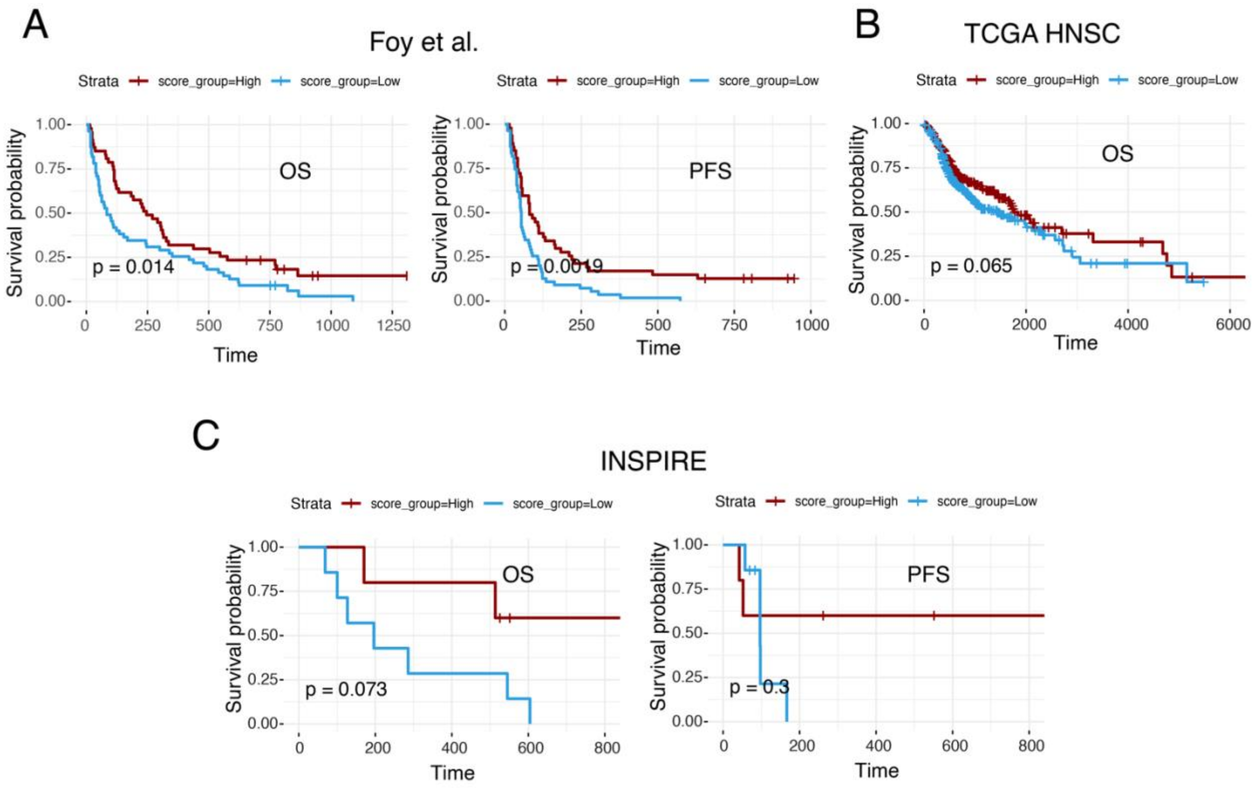

Supplementary Figure 9. Combined effector memory and B cell signature scores associated with HNSC patient survival.

A-C. Kaplan-Meier survival curves for two groups of patients, separated based on LiBIO score, in ICB treatment-naïve (TCGA) (B) and ICB-treated Foy et al. (C) and INSPIRE (D)) cohorts. Survival outcomes are shown for both overall survival (OS) and progression-free survival (PFS). Patients in the high-score group had a more favorable prognosis compared to those in the low-score group.

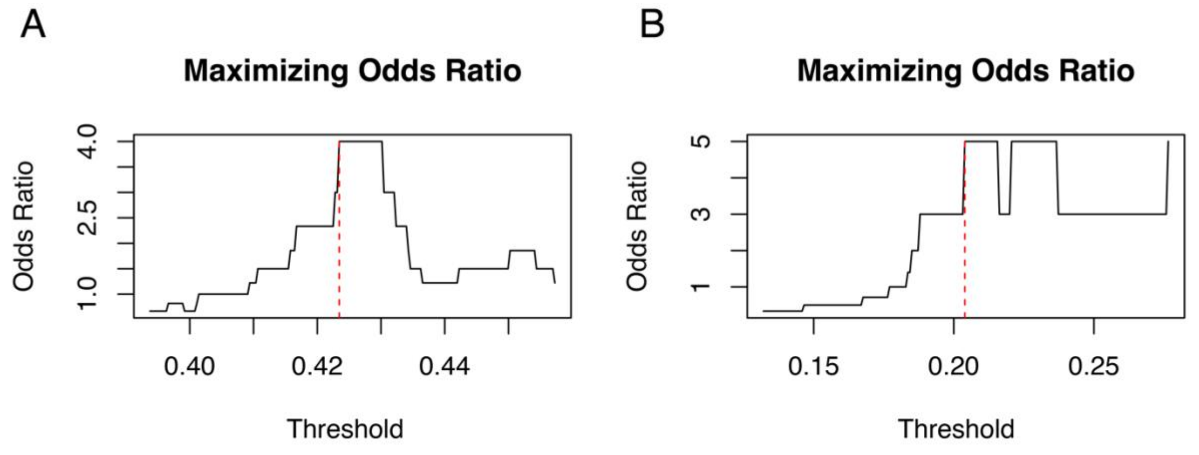

Supplementary Figure 10. Identification of a fixed threshold for the LiBIO score in HNSCC.

A-B. Dynamic changes in odds ratio (OR) across a range of LiBIO score thresholds in single-cell (A) and bulk (B) HNSCC cohorts. Red dashed lines represent the final fixed thresholds used for each data type.

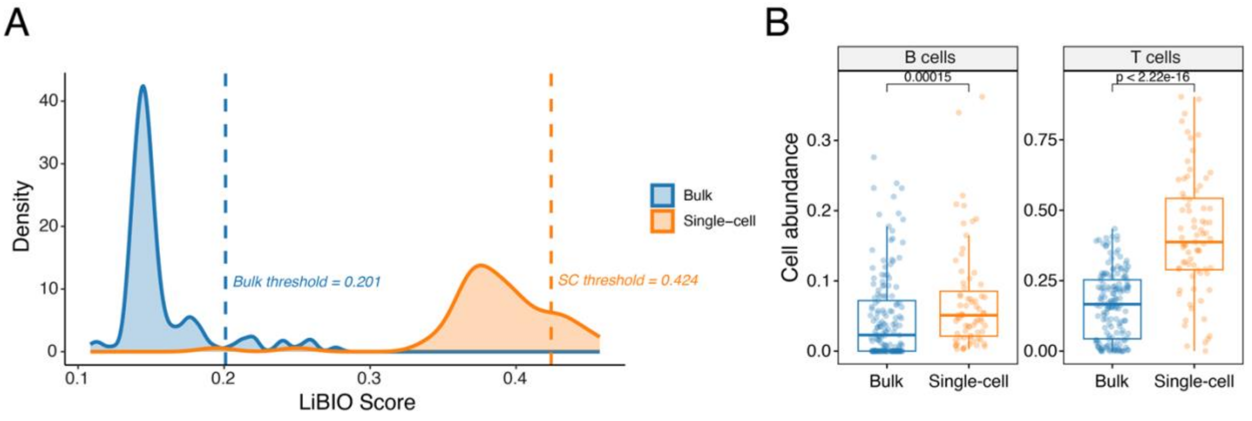

Supplementary Figure 11. Distribution of LiBIO scores and immune cell abundance across bulk and single-cell cohorts.

A. Density plot showing the distribution of LiBIO scores in ICB-treated patient cohorts profiled by bulk RNA-seq (blue) and single-cell RNA-seq (orange). Dashed vertical lines indicate the fixed thresholds used to define responders: 0.201 for bulk data and 0.424 for single-cell data. B. Comparison of B cell and T cell abundance between bulk and single-cell cohorts. The y-axis represents the estimated cell abundance, and each dot corresponds to a single patient. Cell abundances in single-cell cohorts were calculated based on the proportion of annotated cells per sample, while abundances in bulk cohorts were estimated using CODEFACS deconvolution with an HNSCC single-cell reference signature. In box plots, the center line indicates the median; the box spans the interquartile range (IQR, 25th to 75th percentile); whiskers extend to values within 1.5× IQR from the quartiles; and each dot represents one biological replicate (a single patient).

Statistical significance was evaluated using the two-tailed Wilcoxon rank-sum test. Source data are provided as a Source Data file.

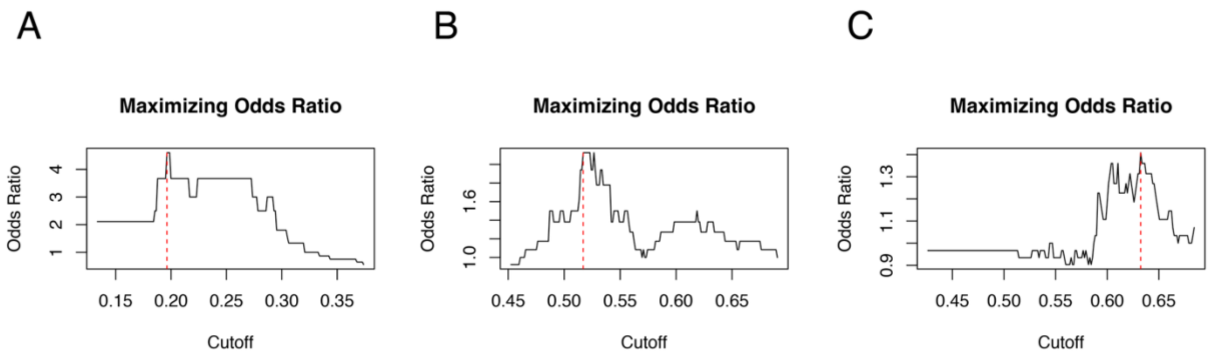

Supplementary Figure 12. Identification of fixed LiBIO score thresholds for melanoma, NSCLC, and breast cancer.

A-C. Dynamic changes in odds ratio (OR) across a range of LiBIO score thresholds in melanoma (A), NSCLC (B), and breast cancer (C) cohorts. For each cancer type, the optimal threshold was selected based on the highest OR for distinguishing responders from non-responders. In cases where multiple thresholds yielded the same maximal OR, the lowest threshold was selected to enhance sensitivity. Red dashed lines represent the final fixed thresholds used for each cancer type.
