# Supplementary material for: Longitudinal liquid biopsy identifies an early predictive biomarker of immune checkpoint blockade response in head and neck squamous cell carcinoma
Source: Nat Commun. 2025 Sep 1;16:8161. doi: 10.1038/s41467-025-63538-4 (PMC12402333; doi:10.1038/s41467-025-63538-4)
Supplement: Supplementary file 3 — Reporting Summary [file 41467_2025_63538_MOESM3_ESM.pdf]

nature portfolio

Corresponding author(s): Kun Wang
J. Silvio Gutkind
Eytan Ruppin1

Last updated by author(s): 07-15-2025

Reporting Summary

Nature Portfolio wishes to improve the reproducibility of the work that we publish. This form provides structure for consistency and transparency in reporting. For further information on Nature Portfolio policies, see our Editorial Policies and the Editorial Policy Checklist.

Statistics

For all statistical analyses, confirm that the following items are present in the figure legend, table legend, main text, or Methods section.

n/a	Confirmed	
☐	☒	The exact sample size (n) for each experimental group/condition, given as a discrete number and unit of measurement
☐	☒	A statement on whether measurements were taken from distinct samples or whether the same sample was measured repeatedly
☐	☒	The statistical test(s) used AND whether they are one- or two-sided<br>*Only common tests should be described solely by name; describe more complex techniques in the Methods section.*
☐	☒	A description of all covariates tested
☐	☒	A description of any assumptions or corrections, such as tests of normality and adjustment for multiple comparisons
☐	☒	A full description of the statistical parameters including central tendency (e.g. means) or other basic estimates (e.g. regression coefficient) AND variation (e.g. standard deviation) or associated estimates of uncertainty (e.g. confidence intervals)
☐	☒	For null hypothesis testing, the test statistic (e.g. F, t, r) with confidence intervals, effect sizes, degrees of freedom and P value noted<br>*Give P values as exact values whenever suitable.*
☒	☐	For Bayesian analysis, information on the choice of priors and Markov chain Monte Carlo settings
☒	☐	For hierarchical and complex designs, identification of the appropriate level for tests and full reporting of outcomes
☒	☐	Estimates of effect sizes (e.g. Cohen's d, Pearson's r), indicating how they were calculated

Our web collection on statistics for biologists contains articles on many of the points above.

Software and code

Policy information about availability of computer code

Data collection	No software was used
Data analysis	All original code used in this study has been deposited in GitHub at https://github.com/wbb1813/Time_series_mouse_ICB and is publicly available as of the date of publication. To ensure reproducibility and provide a permanent citation, the repository has also been archived in Zenodo with the https://doi.org/10.5281/zenodo.15856815. Additionally, Data analysis involved GraphPad Prism version 9 for statistical analyses and R version 4.4.170.

For manuscripts utilizing custom algorithms or software that are central to the research but not yet described in published literature, software must be made available to editors and reviewers. We strongly encourage code deposition in a community repository (e.g. GitHub). See the Nature Portfolio guidelines for submitting code & software for further information.

Data

Policy information about availability of data

All manuscripts must include a data availability statement. This statement should provide the following information, where applicable:

- Accession codes, unique identifiers, or web links for publicly available datasets
- A description of any restrictions on data availability
- For clinical datasets or third party data, please ensure that the statement adheres to our policy

The bulk RNA sequencing data generated in this study have been deposited in the NCBI Gene Expression Omnibus (GEO) under accession code GSE299686 [https://www.ncbi.nlm.nih.gov/geo/query/acc.cgi?acc=GSE299686].

The single-cell RNA sequencing (scRNA-seq) and single-cell T cell receptor sequencing (scTCR-seq) data have been deposited under accession code GSE299683 [https://www.ncbi.nlm.nih.gov/geo/query/acc.cgi?acc=GSE299683].
Source data supporting the findings of this study are provided with this paper.

Research involving human participants, their data, or biological material

Policy information about studies with human participants or human data. See also policy information about sex, gender (identity/presentation), and sexual orientation and race, ethnicity and racism.

Reporting on sex and gender	*Use the terms sex (biological attribute) and gender (shaped by social and cultural circumstances) carefully in order to avoid confusing both terms. Indicate if findings apply to only one sex or gender; describe whether sex and gender were considered in study design; whether sex and/or gender was determined based on self-reporting or assigned and methods used. Provide in the source data disaggregated sex and gender data, where this information has been collected, and if consent has been obtained for sharing of individual-level data; provide overall numbers in this Reporting Summary. Please state if this information has not been collected. Report sex- and gender-based analyses where performed, justify reasons for lack of sex- and gender-based analysis.*
Reporting on race, ethnicity, or other socially relevant groupings	*Please specify the socially constructed or socially relevant categorization variable(s) used in your manuscript and explain why they were used. Please note that such variables should not be used as proxies for other socially constructed/relevant variables (for example, race or ethnicity should not be used as a proxy for socioeconomic status). Provide clear definitions of the relevant terms used, how they were provided (by the participants/respondents, the researchers, or third parties), and the method(s) used to classify people into the different categories (e.g. self-report, census or administrative data, social media data, etc.) Please provide details about how you controlled for confounding variables in your analyses.*
Population characteristics	*Describe the covariate-relevant population characteristics of the human research participants (e.g. age, genotypic information, past and current diagnosis and treatment categories). If you filled out the behavioural & social sciences study design questions and have nothing to add here, write "See above."*
Recruitment	*Describe how participants were recruited. Outline any potential self-selection bias or other biases that may be present and how these are likely to impact results.*
Ethics oversight	*Identify the organization(s) that approved the study protocol.*

Note that full information on the approval of the study protocol must also be provided in the manuscript.

Field-specific reporting

Please select the one below that is the best fit for your research. If you are not sure, read the appropriate sections before making your selection.

[x] Life sciences [ ] Behavioural & social sciences [ ] Ecological, evolutionary & environmental sciences

For a reference copy of the document with all sections, see nature.com/documents/nr-reporting-summary-flat.pdf

Life sciences study design

All studies must disclose on these points even when the disclosure is negative.

Sample size	Sample size was determined based on prior studies and statistical power calculations to ensure sufficient power to detect significant effects.
Data exclusions	No data exclusions were performed; all data points were included in the analysis.
Replication	All experiments were replicated at least twice with consistent results, ensuring reproducibility.
Randomization	Mice were randomly assigned to experimental groups to control for potential confounding variables.
Blinding	Investigators were blinded to group allocation during data collection and analysis to minimize bias.

Reporting for specific materials, systems and methods

We require information from authors about some types of materials, experimental systems and methods used in many studies. Here, indicate whether each material, system or method listed is relevant to your study. If you are not sure if a list item applies to your research, read the appropriate section before selecting a response.

Materials & experimental systems

n/a	Involved in the study
☒	☐ Antibodies
☐	☒ Eukaryotic cell lines
☒	☐ Palaeontology and archaeology
☐	☒ Animals and other organisms
☒	☐ Clinical data
☒	☐ Dual use research of concern
☒	☐ Plants

Methods

n/a	Involved in the study
☒	☐ ChIP-seq
☒	☐ Flow cytometry
☒	☐ MRI-based neuroimaging

Eukaryotic cell lines

Policy information about cell lines and Sex and Gender in Research

Cell line source(s): The 4MOSC1 syngeneic mouse HNSCC cell line was developed by the Gutkind lab and characterized in a prior study (Nature Communications, 2019) line was designed to model tobacco-associated mutational landscapes relevant to head and neck cancer.

Authentication: Cell lines were authenticated through STR profiling by the original developer and confirmed based on consistent morphology and growth characteristics under experimental conditions.

Mycoplasma contamination: All cell lines were tested for mycoplasma contamination and confirmed negative before use in experiments.

Commonly misidentified lines (See ICLAC register): No commonly misidentified cell lines were used in this study.

Animals and other research organisms

Policy information about studies involving animals; ARRIVE guidelines recommended for reporting animal research, and Sex and Gender in Research

Laboratory animals: C57BL/6 mice, aged 6-8 weeks, were used in all experiments. Mice were sourced from Jackson Laboratory and maintained under specific pathogen-free conditions.

Wild animals: This study did not involve wild animals.

Reporting on sex: Both male and female mice were used in the study. Data were disaggregated by sex where relevant, and sex was considered during study design to evaluate any potential sex-based differences. No significant sex-based differences were observed; therefore, combined data are presented.

Field-collected samples: This study did not involve field-collected samples.

Ethics oversight: All animal procedures were approved by the Institutional Animal Care and Use Committee (IACUC) at the University of California, San Diego (UCSD) under protocol number S16200, with Principal Investigator Dr. Joseph Califano. The protocol, titled 'Mouse model for cancer development and drug treatment of cancer,' was approved on 08/23/2022 and is valid until 07/21/2025, covering the use of Mus musculus (mouse) in compliance with federal and institutional regulations.

Note that full information on the approval of the study protocol must also be provided in the manuscript.

Plants

Seed stocks: Not applicable; no plant material was used in this study.

Novel plant genotypes: not applicable

Authentication: not applicable
