# Supplementary material for: Longitudinal liquid biopsy identifies an early predictive biomarker of immune checkpoint blockade response in head and neck squamous cell carcinoma
Source: Nat Commun. 2025 Sep 1;16:8161. doi: 10.1038/s41467-025-63538-4 (PMC12402333; doi:10.1038/s41467-025-63538-4)
Supplement: Supplementary file 4 — Description of Additional Supplementary Files [file 41467_2025_63538_MOESM4_ESM.pdf]

Description of Additional Supplementary Files

File name: Supplementary Data 1

Description: Signature genes of effector memory $CD8^+$ T cells (T_{em}) and B cells were identified using single-cell data from the second timepoint (Day 9). Marker genes for T_{em} and B cells were determined by comparing gene expression between responder and non-responder mice, with selection based on a false discovery rate ($FDR \leq 0.01$) and fold change ≥ 1.5.
